# Zoonotic parasites carried by invasive alien species in China

**DOI:** 10.1186/s40249-018-0512-6

**Published:** 2019-01-09

**Authors:** Guang-Li Zhu, Yi-Yang Tang, Yanin Limpanont, Zhong-Dao Wu, Jian Li, Zhi-Yue Lv

**Affiliations:** 1 Zhongshan School of Medicine, Sun Yat-sen University, Guangzhou, 510080 China; 2 Fifth Affiliated Hospital, Sun Yat-sen University, Zhuhai, 519000 Guangdong China; 3 Key Laboratory of Tropical Disease Control, Ministry of Education, Sun Yat-sen University, Guangzhou, 510080 China; 4 Provincial Engineering Technology Research Center for Biological Vector Control, Guangzhou, 510080 China; 5 Faculty of Tropical Medicine, Mahidol University, Bangkok, 10400 Thailand

**Keywords:** Invasive alien species, Zoonotic parasite, China

## Abstract

**Background:**

The invasive alien species may lead to great environmental and economic crisis due to its strong capability of occupying the biological niche of native species and altering the ecosystem of the invaded area. However, its potential to serve as the vectors of some specific zoonotic pathogens, especially parasites, has been neglected. Thus, the damage that it may cause has been hugely underestimated in this aspect, which is actually an important public health problem. This paper aims to discuss the current status of zoonotic parasites carried by invasive alien species in China.

**Main body:**

This review summarizes the reported zoonotic parasites carried by invasive alien species in China based on the Database of Invasive Alien Species in China. We summarize their prevalence, threat to human health, related reported cases, and the roles of invasive alien species in the life cycle of these parasites, and the invasion history of some invasive alien species. Furthermore, we sum up the current state of prevention and control of invasive alien species in China, and discuss about the urgency and several feasible strategies for the prevention and control of these zoonoses under the background of booming international communications and inevitable globalization.

**Conclusions:**

Information of the zoonotic parasites carried by invasive alien species neither in China or worldwide, especially related case reports, is limited due to a long-time neglection and lack of monitoring. The underestimation of their damage requires more attention to the monitoring and control and compulsory measures should be taken to control the invasive alien species carrying zoonotic parasites.

**Electronic supplementary material:**

The online version of this article (10.1186/s40249-018-0512-6) contains supplementary material, which is available to authorized users.

## Multilingual abstracts

Please see Additional file 1 for translations of the abstract into the five official working languages of the United Nations.

## Background

Invasive alien species (IAS) are non-native species introduced deliberately or unintentionally outside their natural habitats where they become established, proliferate and spread in ways that cause damage to a wide range of interests [1].

The screening and monitoring of IAS is of urgent requirement due to its potential capability to damage ecology, economy and most alarmingly, public health. Probably because of the absence of its predators and the great adaptability to different environments, IAS invade and occupy the ecological niche of the native and destructs local biodiversity. Giving its catastrophic nature, IAS have already been regarded as the second greatest factor of species extinction and endangerment [2]. Additionally, the economic loss associated with IAS sums up to about 120 billion dollar/year in the United States of America [3]. The economic loss caused by IAS was estimated nearly 19.8 billion CNY in China [4]. However, the risks from IAS to public health have been neglected. The IAS themselves can impair human health directly. For example, a sting of red imported fire ant (*Solenopsis invicta*) can cause severe anaphylactic reaction to human beings [5]. In addition, as either the vector or reservoir host of parasites, IAS will carry not only the native parasites, but also the non-native ones, which may threaten the public health, and delay the diagnosis and treatment simply due to the unfamiliar of the native doctors to the pathogens.

With a vast territory of approximate 9.6 million square kilometers covering almost all ecosystems in the world, there are always suitable habitats for many non-native species in China. In addition to a long history of the introduction of non-native species, the rapid development of economy, technology and transportation system since Chinese Economic Reform has also greatly increased the risk of biological invasion by non-native species, accompanied with the increasing in population density and trading activities.

In 2017, according to the Database of Invasive Alien Species in China (www.chinaias.cn) published by Center for Management of Invasive Alien Species, Ministry of Agriculture, a total of 270 invasive alien animal species have been recorded, including one myxozoa, 14 moss animals, one annelid, one echinoderm, 17 nematodes, 20 mollusks, 153 arthropods (140 insects), six ascidians, 34 fishes, four amphibians, three reptiles, six birds, 10 mammals.

About two thirds of the invasive alien species were introduced intentionally [6]. Before the concept of biological invasion were widely accepted, the species introduction was mainly focused on socioeconomic benefit. It was not until middle of 1990s when several Chinese scientists’ papers introduced the concept and impact of IAS that the government and the public began to take actions to deal with the problem of IAS. For such a long time, the impact of IAS had been neglected, especially their impact on public health [7]. As vectors of native pathogens, expanding IAS may lead to an increase of infection rate. [8]. What’s worse, the novel non-native pathogens introduced by these IAS may even cause an outbreak in the native populations. More attention to these parasites should be aroused [9]. This paper aims to discuss the current knowledge of zoonotic parasites carried by invasive mollusks, arthropods and vertebrates in China, mainly focusing on the category, clinical aspects and administration and international cooperation to prevent and control the problems.

### Invasive alien mollusk harboring zoonotic parasites

Most mollusks IAS capable of carrying parasites and transmitting parasitic disease are freshwater snails and human may be infected by ingesting raw or undercooked snail. The most common species include the *Pomacea canaliculata*, *Achatina fulica* Bowditch and *Biomphalaria straminea* (Dunker).

#### *Pomacea canaliculata*

*P. canaliculata*, which is commonly known as golden apple snail, originated from Argentina, was first introduced into Taiwan in the 1970s and mainland China in 1981 [6] as edible material [10]. With its wide tolerance toward different environments and high reproduction rate, the snail exhibits the capability to establish wild populations rapidly in new habitats [11]. And it has already spread to at least 16 PAM (province/autonomous region/municipality) in China including Taiwan, Guangdong, Guangxi, Fujian, Zhejiang, Jiangsu, Shanghai, Hubei, Jiangxi, Liaoning, Beijing, Anhui, Sichuan, Chongqing, Gansu, Tianjin [12].

Based on the biology of the snail and the meteorological database of 670 weather sites distributed all across China during 1951–1980, the researchers worked out a model to predict the potential habitat for the species. The model suggested that about 52.1% regions represented by the above weather sites (about 2 563 000 km^2^) were vulnerable to the colonization of the *P. canaliculate* [13]*.* Considering the future climate change and the inevitable trend of global warming, an area of approximately 378 700 km^2^ is expected to be occupied by *P. canaliculata* from the 2020s to the 2030s, in which the distribution could move further northeast, even covering the Huaihe River valley [12]. The *P. canaliculata* poses a huge threat to the agriculture industry as well. It is reported to be potential pest for 15 important economic agricultural crops in China [14]. Also, *P. canaliculata* is able to spread rapidly into wetland [15] and other freshwater ecosystems, resulting in severe habitat modification and a decrease of local biodiversity [16].

Apart from its direct destruction to the ecosystem, *P. canaliculata* is able to impair the human health directly as the intermediate host of several parasites such as *Echinostoma revolutum*, *Angiostrongylus cantonensis* and *Gonathostoma spinigerum* which respectively cause echinostomiasis, angiostrongyliasis and gnathostomiasis [17].

Angiostrongyliasis is the most common cause of eosinophilic meningitis in southeast Asia and the Pacific basin [18] and usually results from consuming the undercooked snail or other mollusk which carry the infectious larvae of *A. cantonensis* [19]*.* The first case reported came from Taiwan in 1945 [20], and was first discovered in Guangdong Province (mainland China) in 1984 [21]. From 2000 to 2006, six outbreaks has attracted the public’s attention [22], among which the most serious one occurred in Beijing in 2006 involving 160 infected and 100 hospitalized [23].

Echinostomiasis is caused by orally intaking the metacercaria of the family Echinostomatide via the raw or undercooked infected snails [24]. The number of involved provinces in mainland China had reached eight by 1994, including Heilongjiang, Sichuan, Yunnan, Zhejiang, Fujian, Hubei, Guangdong, Anhui [25]. In some coastal provinces like Fujian and Guangdong where edible snails are easily accessible, the prevalence of the disease even reaches 5.0% [26].

*G. spinigerum* is another parasite that may be carried by *P. canaliculata* [27]. Nevertheless, it is unusual to find a patient infected after consumed the snail. Almost all the cases reported in China got infected by the raw or undercooked fish and the incidence is rather low. From 1919, when index case in China came out to 2011, only 73 cases covering 16 PAM were reported, among which Zhejiang, Shanghai, Fujian, Guangdong had higher incidence [28].

#### *Achatina fulica*

Another invasive mollusk capable of transmitting parasites is the *A. fulica*, also known as giant Africa snail. It is firstly discovered in Xiamen, Fujian Province in the plant carried by a Singapore overseas Chinese in 1931 [29]. After settling down in Xiamen, it began to scatter in southern part of mainland China including Guangdong, Hainan, Guangxi. It invaded Hong Kong, Macau in 1932 and Taiwan in 1933 [30]. The snail in Yunnan was introduced in 1979 from Vietnam [31].

Its various trophic preference and high reproductive ability make it pest for many agricultural crops and ornamental plants. The rapid establishment and expansion can jeopardize the biodiversity and impair the local plants and animals [32]. Besides, it is capable to carry many pathogens which harm human health directly such as *A. cantonensis* [33]. Due to its biological characteristics, *A. fulica* is even more vulnerable to the infection of *A. cantonensis* than *P. canaliculata*. A survey conducted in Jiangmen, Guangdong Province indicate that the infection rate of *A. fulica* and *P. canaliculata* by *A. cantonensis* reached 41.9 and 1.8%, and the degree of infection was 1–928 per snail and 1–29 per snail respectively [34]. But the threaten caused by *A. fulica* are not as severe as the *P. canaliculata* because of its terricolous feature [22]. *A. fulica* can be infected by the *A. costaricensis* larvae under laboratory condition, so the snail can be the intermediate host of *A. costaricensis* as well [35]. However, no relevant case has been reported in China yet.

## *Biomphalaria straminea*

*B. straminea* attracts attention as the intermediate host of *Schistosoma mansoni*, which may lead to schistosomiasis [36]. The *B. straminea*, originated from South America, was first discovered in Hong Kong in 1974 and was thought to be introduced abroad. Later in 1981, it appeared in a pond in the Luohu District of Shenzhen City, which was the first report in mainland China [37]. By 2013, the snail had spread in large range of Shenzhen and overspread to the surrounding regions such as Dongguan and Huizhou in Guangdong Province [38]. While no infection of *B. straminea* by *Sc. mansoni* in China has been detected, *Sc. mansoni* infection in the returnees of China from Africa is increasing in recent years [39]. Because Shenzhen and surrounding regions are key area of labor exporting with large population mobility and numerous international communications, some experts presumed that China is now equipped with the objective condition for the prevalence of *Sc. mansoni* [40]*. B. straminea* was also found capable of carrying the larvae of *A. cantonensis* in Brazil, which may cause angiostrongyliasis but there is no such a case reported in China yet [41].

The information concerning the invasive alien mollusks and the parasites carried by them worldwide is listed in Table 1.

**Table 1:** Zoonotic parasites carried by invasive alien mollusks

Species	Common name	Parasites	Reference
*Mytilus galloprovincialis* Lamarck	Mediterranean mussel	*Cryptosporidium parvum*	[111]
		*Giardia duodenalis*	[112]
		*Toxoplasma gondii*	[113]
*Dreissena polymorpha*	Zebra mussel	*Acanthamoeba* spp.	[114]
		*Cryptosporidium* spp.	[115]
		*G. duodenalis*	[116]
*Pomacea canaliculata*	Golden apple snail	*A. cantonensis*	[19]
		*Echinostoma revolutum*	[24]
		*Gonathostoma spinigerum*	[27]
*Achatina fulica* Bowditch	Giant African snail	*Angiostrongylus costaricensis*	[35]
		*A. cantonensis*	[33]
*Biomphalaria straminea* Dunker		*Schistosoma mansoni*	[36]
		*A. cantonensis*	[117]

### Invasive alien arthropods harboring zoonotic parasites

#### Invasive alien crustaceans harboring zoonotic parasites

*Procambarus clarkii,* also commonly acknowledged as the red swamp crayfish or red swamp crawfish, is the native species of North America [42]. It was introduced to Japan in 1918, and then brought into Nanjing, China in the 1930s during the Second World War from Japan [43]. Nowadays, it expanded all over China as an important food resource [44] and the sales are becoming increasingly larger [45], but it is not completely safe. In 1952, *Paragonimus westermani* was first discovered in *P. clarkii* in Japan [46]. People may get infected through the intake of the undercooked crayfish [47]. The paragonimiasis is a zoonotic disease prevailing in 25 PAM at varied extent and has higher incidence in southwest China including Sichuan, Chongqing and Yunnan [48]. More than 100 counties in Sichuan Province had reported cases through past 50 years and the incidence shows an upward trend because of the changing in the lifestyle and diet [49]. However, there has been no definite report regarding to *P. clarkii* as the cause of the paragonimiasis in China so far. Patients commonly got infected by consuming the undercooked *Potamon* spp., or some freshwater snails or through direct contact with the contaminated water. This could be explained by the fact that most edible *P. clarkii* in China comes from the aquafarm where the safety is guaranteed.

#### Invasive alien insects harboring zoonotic parasites

Most invasive alien insects in China have a closer relationship with plants instead of animals except *Blattella germanica, Periplaneta Americana, Periplaneta australasiae, S. invicta* and so on.

##### *Tribolium castaneum*

The *Tribolium castaneum* (Herbst), also known as red floor betel, is recognized as the pest of stored grain. The species distributes worldwide in 6 continents except the Antarctica according to the Bureau of Entomology and Plant Quarantine, United States Department of Agriculture [50]. It was reported that the betel could only be found in 4 provinces in China by the 1960s. The species made its way through the transportation and trading of grain food. In consideration of the tradition of food allocation and transportation around China, researchers assumed the *T. castaneum* as one of the IAS [51]. Nowadays, this specie has invaded 23 PAM of China, while no evidence found in Shanghai, Shanxi, Shaanxi, Qinghai, Gansu, Xinjiang, Taiwan, Guizhou and Tibet [52].

*T. castaneum* is experimentally proved as the potential intermediate host for *Pseudanoplocephala crawfordi*, which may cause psudanoplocephalosis [53]. *P. crawfordi* inhabits in the small intestines of many mammals including the swine, mouse and human. The patients usually take on some abdominal symptoms [54]. The prevalence of the parasites in the swine of China can reach 15.2–91.6% which makes it a big threat to the livestock industry [55]. Twenty-one cases [56] related to this parasite had been reported in Henan [56], Liaoning [53] and Shaanxi [57] until 2016. Patients can be infected by undertaking insanitary food contaminated by the *T. castaneum* or eating the *T. castaneum* carrying the cysticercoids directly.

##### Cockroaches

Cockroach is one of the most common synanthropic insects in the world, especially in the areas with bad sanitary condition. In China, before 1980s, the dominant species of city cockroaches are *P. americana*, while the distribution of *B. germanica* is limited to several cities. Later, the invasive *B. germanica* replaced *P. americana* to be the dominant species in city and spread nationwide rapidly [58].

Because cockroaches usually feed on human feces, they may transmit various zoonotic parasites mechanically. An investigation in Taiwan revealed a high infection rate, 25.0% in *P. americana* and 10.0% in *B. germanica*, by *Entamoeba histolytica* or *Entamoeba dispar* respectively [59]. Besides, cockroaches can induce allergic reaction in some people directly [60].

More zoonotic parasites carried by the invasive alien insects documented worldwide are listed in Table 2.

**Table 2:** Zoonotic parasites carried by invasive alien insects

Species	Common name	Parasites	Reference
*Blatella germanica*	German cockroach	*Ascariasis lumbricoides, Enterobius vermicularis, Entamoeba histolytica, E. dispar*, *G. lamblia,* hookworm, *Hymenolepis nana, Strongyloides stercoralis, Sarcocystis muris, Trichuris trichiura, Taenia* spp., *T. gondii*	[118]
*Periplaneta americana*	American cockroach	*Moniliformis moniliformis, Diplogaster* sp.	[119]
		*A. lumbricoides, Balantidium coli, Entamoeba coli, E. histolytica, E. vermicularis,* hookworm, *T. trichiura*	[120]
		*S. muris, T. gondii*	[121]
		*Schistosoma haematobium, Sc. mansoni*	[122]
*Tribolium castaneum* Herbst	Red flour beetle	*Hymenolepis diminuta*	[123]
		*Pseudanoplocephala crawfordi*	[53]
		*Steinernema feltiae*	[124, 125]
*Cydia pomonella*	Codling moth	*S. feltiae*	[126]

### Zoonotic parasites carried by invasive alien vertebrates

#### Zoonotic parasites carried by invasive alien fishes

Unlike other IAS, most invasive alien fishes were initially introduced intentionally. Some of them escaped into natural water, spread rapidly and became the dominant species in local waters. More than 65 species of fishes have been introduced into China [61]. Most of them were introduced for aquaculture to increase the food supply. Since 1957, when *Oreochromis mossambicus* was introduced into China from Vietnam firstly, more species of tilapias had been introduced, including *O. niloticus, Tilapia zillii.* Now *O. mossambicus* has widely distributed in waters of South China and established wild populations [62]. Some species were introduced as ornamental species, like *Hypostomus plecostomus.* Some are introduced for biological control. The best-known example is the mosquitofish, *Gambusia affinis,* which was thought to be effective in controlling mosquitoes. However, it was proven that its effect in controlling mosquitoes did not differ from local species significantly. On the contrary, it invaded local waters by feeding on fry. Initially introduced into China from Philippine as early as 1927, mosquitofish have replaced *Oryzias latipes* and *O. Curvinotus* to be local dominant species in waters of southern China [63].

Fishes are important source of zoonotic parasites. Almost all fish-borne parasitic zoonoses have been acquired by consuming raw or undercooked fish product like sushi, sashimi and so on. As is estimated by the World Health Organization (WHO), more than half billion people are exposed to the risk of these diseases [64]. The rapidly proliferating IAS may serve as a dramatically expanding source of zoonotic infection or in another way assist the transmission of these zoonotic parasites to other fishes.

#### Fish-borne zoonotic trematodes

Fish-borne zoonotic trematodes mainly consist of four families, including Opisthorchiidae, Heterophyidae, Nanophyetidae, and Echinostomatidae [65]. For these trematodes, fishes mainly serve as the second intermediate host [66]. Fish-borne zoonotic heterophyids mainly include *Heterophyes* (*H. heterophyes*, *H. dispar*), *Haplorchis* (*H. taichui*, *H. pumilio*, *H. yokogawai*, and *H. vanissimus*), *Metagonimus* (*M. minutus*, *M. yokogawai* and *M. takahashii*), *Centrocestus* (*C. formosanus*), *Procerovum* (*P. varium*) [67]. Nine heterophyid trematodes have been reported in China, mainly in Southern China, where the patient infected with heterophyid was usually coinfected with *Clonorchis sinensis* due to the habit of eating raw fish [68]. Information of heterophyids infection in invasive alien fishes in China was displayed in Table 3. These zoonotic parasites were endemic in these regions correspondingly. In Longhai City and Nanjing County of Fujian Province, the infection rate of the local population is 0.3% (13/3867) [69]. A survey in Guangxi, where people has a habit of consuming raw fish, revealed that the infection rate of *H. taichui* reached about 24.3% there. Surprisingly, in one investigating site, *H. taichui* infection accounts for 90.5% of people with trematode infection. The most commonly consumed raw fish were *C. carpio* and *C. auratus* [70]. Besides, although experimental human infection of *C. formosanus* has been reported, natural human infection has never been recorded [71].

**Table 3:** Infection rate of heterophyids in invasive alien species in different cities in China

Parasites	Species of local invasive alien species host	Location	Infection rate	Reference
*Haplorchis pumilio*	*Cyprinus carpio*, *Carassius auratus*	Longhai City and Nanjing County of Fujian Province	36.3%	[69]
*H. taichui*	*C. auratus*	Yangshuo County, Nanning City, Mashan County, Fusui County and Binyang County in Guangxi	61.9%	[68]
	*C. carpio*		42.9%	
	*Tilapia nilotica*		0	
	*Cirrhinus molitorella*		0	
	*Gambusia affinis*		–	
*H. pumilio*	*C. auratus*		81.0%	
	*C. carpio*		28.6%	
	*T. nilotica*		0	
	*C. molitorella*		0	
*Centrocestus formosanus*	*G. affinis*, *C. carpio*, *C. auratus*	Guangdong, Fujian, Guangxi, and Taiwan	–	[127]

Among family Opisthorchiidae, three liver flukes, *C. sinensis*, *Opisthorchis felineus*, and *O. viverrini* are the most problematic zoonotic parasites [72]. For the Chinese liver fluke *C. sinensis*, a survey in 27 endemic PAM in China, revealed a high prevalence of about 2.4%, about 12.5 million people [73]. The selectivity on second intermediate host of *C. sinensis* is low. Sixty eight species of freshwaters as latent second intermediated host of *C. sinensis* have been reported in China, including *C. auratus*, *C carpio*, *O. mossambica* and so on [74, 75]*.*

##### Fish-borne zoonotic cestodes

Fish-borne zoonotic cestodes are mainly composed of *Diphyllobothrium* (*D. latum* and *D. dendriticum*) [76]. A total of 16 cases of *D. latum* infection through consuming raw fish have been reported in China, but no detailed information about the species of fish was documented [77]. *D. latum* infection has been recorded in *Oncorhynchus mykiss, Salvelinus forntinalis, Stizostedion vitreum, Perca flavescens* worldwide as listed in Table 3.

##### Fish-borne zoonotic nematodes

Fish-borne zoonotic nematodes consist of parasites of family Gnathostomatidae (*Gnathostoma* spp.), family Anisakidae (*Anisakis*), family Capillariidae (*Capillaria philippinensis*), family Dioctophymatidae (*Eustrongylides* spp.). Among them, the most common species infecting humans are *Gnathostoma* spp., *Anisakis* spp. and *Pseudoterranova* spp. [78]. More than 50 cases of gnathostomiasis have been reported in 16 PAM of China. Most common infection way of *Gnathostoma* spp. is also consumption of raw freshwater fishes, including *C. carpio*, and *C. auratus* [79]*.*

Among these fish-borne zoonotic parasitic infection cases, most of them were wild-caught fishes, while only a few were farm-raised fishes, which have been neglected for a long time. In a research conducted in Guangdong Province, where almost half of the tilapia in China is produced, zoonotic parasites of family Heterophyidae and Echinostomatidae were detected in the cultured tilapias [80].

The zoonotic parasites carried by more invasive alien fishes of China have been listed in Table 4, which is documented worldwide. Although associated information in China is limited, this table provides an insight into the potential risks of consuming these fishes in China, especially under the circumstance that infection rate in some populations, in fact is high but ignored as illustrated above, which may be attributed to the fact that most infections only cause light symptoms like diarrhea or other gastrointestinal disorders. Since most invasive alien fishes were introduced for aquaculture, more attentions should be paid to zoonotic parasites carried by these fishes.

**Table 4:** Zoonotic parasites carried by invasive alien fishes

Invasive alien species	Common name	Parasites	Reference
*Oncorhynchus mykiss*	Rainbow trout	*Diphyllobothrium dendriticum*	[76]
		*D. latum*	[128, 129]
		*Cryptosporidium* sp.	[130]
*Salvelinus forntinalis*	Brook trout	*D. dendriticum, D. latum*	[131]
*Piaractus mesopotamicus* Holmberg		*Eustrongylides* sp.	[78]
*Gambusia affinis*	Mosquitofish	*Centrocestus formosanus*	[127, 132]
		*Haplorchis taichui*	[127]
		*H. yokogawai*	[67]
		*H. pumilio*	[127, 133]
*Stizostedion vitreum*	Walleye	*D. latum*	[129]
*Perca flavescens*	Yellow Perch	*D. latum*	[129, 134]
*Lucioperca lucioperca*	Pike-perch	*Eustrongylides* sp.	[135]
		*Anisakis simplex*	[136]
*Cichlasoma managuense, Parachromis managuensis*		*Gnathostoma* sp.	[137]
*Oreochromis niloticus*	Nile tilapia	*H. pumilio, H. taichui, Procerovum varium, Metagonimus* spp., *C. sinensis*	[80]
		*Gnathostoma* sp.	[138]
		*Cryptosporidium* spp., *Prohemistomum* spp.	[139]
		*C. formosanus*	[140]
*Oreochromis nilotica*	Red tilapia	*C. philippinensis*	[141]
		*H. yokogawai*	[67]
*Tilapia zillii*		*Capillaria* sp., *Diphyllobothrium* sp., *H. nana, Trichostrongylus* sp.	[142]
*Oreochromis* sp.		*Cryptosporidium* sp.	[130]
*Oreochromis mossambicus*	Black tilapia	*C. sinensis*	[75, 143]
		*Gnathostoma* sp.	[138]
*Clarias* spp.		*H. pumilio*	[144]
*(Clarias lazera* Cuvier & Valenciennes *Clarias macrocephalus* Günther *Clarias batrachus* L*.)*		*H. taichui* (for *C. batrachus*)*, H. yokogawai* (for *C. batrachus*)	[145]
		*Gnathostoma spinigerum*	[146, 147]
*Pangasius* sp*.*		*H. pumilio*	[144]
		*C. formosanus*	[148]
*Siluris glanis*	European catfish	*Eustrongylides* sp.	[135]
*Ictalurus punctatus*	Channel catfish	*Eustrongylides* sp.	[149]
*Channa striata*	Snakehead fish	*H. pumilio, H. taichui, O. viverrini, Procerovum* sp.	[150]
		*G. spinigerum*	[147]
*Channa micropeltes*		*Gnathostoma* sp.	[151]
*Lates calcarifer*	Asian seabass	*Cryptosporidium* sp.	[130]
*Cirrhina mrigala/Labeo rohita*	Mrigal/Rohu	*C. formosanus, C. sinensis, H. pumilio, H. taichui, H. yokogawai, P. varium*	[145, 152]
*Cyprinus carpio var. specularis.*	Mirror carp	*C. sinensis*	[75, 153]
*Cyprinus carpio var. Mirror splittered*		*C. formosanus, H. pumilio, H. yokogawai*	[145]
		*O. viverrini*	[154]
		*H. taichui*	[127]
*Carassius auratus* Cuvieri Temminck & Schlegel	Japanese crucian carp	*C. sinensis, Centrocestus armatus, Metagonimus takahashii, Metagonimus* spp.	[127]
		*Haplorchis* spp.	[155]
		*Metorchis orientalis*	[65]
		*O. felineus*	[72]
		*O. viverrini*	[156]
		*H. yokogawai*	[67]
*Chalcalburnus chalcoides*		*Anisakis* sp.	[157]

#### Zoonotic parasites carried by invasive alien amphibians

Invasive alien amphibians consist of *Rana catesbeiana, R. heckscheri, R. grylio, R. tigrine, Trachemys scripta elegans* and *Mauremys mutica*.

*R. catesbeiana*, bullfrog, was first introduced into China in 1959. Along with the expansion of the farming of bullfrog, some escaped into wild environments and established wild populations, but most wild populations are still small, which makes it hard to capture bullfrog in wild.

Frogs are the second intermediate host of *Spirometra mansoni*. A reported infection rate of *Sp. masoni* in bullfrog in Japan and USA were 43.0 and 1.7% respectively [81, 82]. However, in the investigation in Zhejiang Province and Guangzhou City, Guangdong Province, although infection rate is high in the wild frogs, like *R. tigrina rugulosa* and *R. limnocharis*, no *Sp. mansoni* infection was found in the farm-raised bullfrogs [83–85]. It may be associated with the absence of first intermediate host, cyclops or definitive host cat or dogs in the farming process. But high infection rate in wild frogs still alarms us that the wild bullfrog populations should never be neglected even though they are rarely captured. In addition, *Gnathostoma nipponicum* has also been detected in bullfrog in Japan [86].

#### Zoonotic parasites carried by invasive alien birds

No information about the zoonotic parasites carried by invasive alien birds is available in China, but there are a few reports about these parasites worldwide, which are listed in Table 5.

**Table 5:** Zoonotic parasites carried by invasive alien birds

Species	Common name	Parasites	Reference
*Psittacula krameri*	Ring-necked parrot	*Cryptosporidium meleagridisin*	[158]
*Branta canadensis*	Canadian goose	*Cryptosporidium parvum*	[159, 160]
		*Toxoplasma gondii*	[161]
		*C. hominis*	[162]
		*Giardia* sp.	
		*Trichobilharzia* spp.	[163]

#### Zoonotic parasites carried by invasive alien mammals

Invasive alien mammals consist of *Macaca fascicularis* and nine species of rodents, including 6 species of rats*.* Rats are the most common rodents in the communities and thus play a significant role in human health as a vector of many zoonotic pathogens, especially in the areas with high temperature and poor sanitation.

Invasion history of *Rattus norvegicus, R. rattus* and *Mus musculus* can’t be traced because their invasion was far before the foundation of the People’s Republic of China, PRC and the beginning of nationwide monitoring of rodents. Thus, with such a long history and great adaptability to all kinds of environments, they have spread all over China. A survey in Zhejiang Province showed that dominant species of rodents are *M. musculus* and *R. norvegicus* indoor and outdoor respectively [87]. And *M. musculus* and *R. norvegicus* ranked 2nd and 3rd in number of indoor rodents in Shanghai, respectively [88]. They might be imported through ocean shipping [89]. And modern transportation may accelerate their invasion. Before the railway opened to traffic in Xinjiang Uygur Autonomous Region, there is no record of *R. norvegicus.* Since 1975 when *R. norvegicus* was first captured in a train in Xinjiang, *R. norvegicus* have dramatically expanded in local rodent population and became the dominant species in 1989 [90]. Another species of invasive alien rodent, *Ondatra zibethicus,* spread into China from Union of Soviet Socialist Republics through the rivers at northwestern and northeastern border [91]. Besides, *Myocastor coypus* was initially introduced and farmed as fur animal. But as the fur quality dropped in the farmed *M. coypus* in southern China, they were abandoned and became wild [92].

##### Heterophyids

*H. nana* and *H. diminuta* are the common zoonotic intestinal parasites of wild rats. Xinjiang is a high incidence area of *H. nana* infection, where some regions can reach even 9.2%. In these areas, *H. nana* infection rate is relatively high in *R. norvegicus* and *M. musculus* [93]. In addition, an average infection rate of 0.02 and 0.01% for *H. diminuta* and *H. nana*, respectively was reported in an investigation in Yunnan Province, while it was 0.1 and 0.2% in Henan Province [94, 95].

##### *Angiostrongylus cantonensis*

*A. cantonensis* was first identified in the lung of *R. rattus* and *R. norvegicus* in Guangzhou in 1933. It’s thought that *R. rattus* and *R. norvegicus* are the most common definitive host of *A. cantonensis*, which is indispensable for the establishment of its foci [96]. The presence of this parasite in rats indicates that this region is endemic. *R. norvegicus*, *R. tanezumi* had been reported to harbor this parasite in China [97].

##### *Babesia microti*

There are many natural foci of *Babesia microti* in China and several sporadic cases of human infection have been reported in 8 PAM [98]. A case of human babesiosis was reported in Zhejiang Province in 2012, and the local investigation found *B. microti* infection in two *R. tanezumi* and one *R. norvegicus* [99]*.*

##### *Echinococcus multilocularis*

*Echinococcus multilocularis* is endemic in northwest China, mainly Xinjiang, Ningxia, Gansu, Sichuan and Qinghai, where there are still many semi-nomads. Over 1000 cases of alveolar echinococcosis caused by *E. multilocularis* had been reported in China from 1956 to 2005 [100]. In Xinjiang, *E. multilocularis* was detected in *R. norvegicus* and *M. musculus* [100, 101]*.* However, the infection rate of *E. multilocularis* might have been underestimated a lot, as about 200 cases were reported just in the First affiliated hospital of Xinjiang Medical University [102]. The low infection rate reported may be attributed to the underdevelopment of those regions and limit of their technology of diagnosis.

##### *Capillaria hepatica*

*C. hepatica* is a rare zoonotic parasite with only three cases of human infection reported in China [103]. So far, only one case of *C. hepatica* infection in human has been reported in Fujian Province. However, a local investigation revealed a high infection rate, 10.8 and 7.7% respectively in *R. norvegicus* and *M. musculus* [104]*.*

##### *C. sinensis*

*O. zibethicus, R. norvegicus* and *M. musculus* can serve as the reservoir host of *C. sinensis* in China [105].

As being estimated, rodents are vectors of at least 60 zoonotic diseases [106]. While researches on the zoonotic parasites carried by these alien invasive rodents are limited in China, many globally reported parasites are not detected, which are listed in Table 6.

**Table 6:** Zoonotic parasites carried by invasive alien mammals

Invasive alien species	Common name	Parasites	Reference
*Rattus norvegicus*	Wild brown rats	*Angiostrongylus cantonensis*	[19, 106, 164–167]
		*Echinococcus multilocularis*	[168]
		*Capillaria hepatica, Hymenolepis diminuta, Taenia taeiaeformis*	[169]
		*Schistosoma mansoni, Moniliformis moniliformis*	[170]
		*H. nana, Giardia* sp., *Cryptosporidium* sp., *Entamoeba* sp.	[171]
		*C. parvum*	[172]
		*Entrobius* spp., *Trichuiris* spp.	[173]
		*Leishmania major*	[174]
		*L. infantum*	[175]
		*Toxoplasma gondii*	[161]
		*Trichinella spiralis, T. britovi, T. pseudospiralis*	[176]
		*Toxocara canis*	[177]
		*Gongylonema pulchrum*	[178]
		*Syphacia muris*	[179]
		*Sarcocystis orientalis*	[180]
		*Paragonimus westermani, Plagiorchis potamonides, Echinostoma ilocanum, Raillietina madagascariensis*	[181]
		*S. japonicum*	[182]
*Rattus rattus*	House rat/Roof rat	*Rictularia* sp., *H. diminuta, M. moniliformis*	[168]
		*C. parvum*	[183]
		*Fasciola hepatica*	[184]
		*Taenia taeniaeformis*	[185]
		*Leishmania donovani donovani, L. donovani infantum*	[186]
		*A. cantonensis*	[166]
		*G. pulchrum*	[178]
		*T. britovi*	[176]
		*T. gondii*	[187]
		*Sc. mansoni*	[188]
		*Sarcocystis* sp.	[180]
		*Babesia microti*	[98]
		*T. spiralis, Entamoeba coli, Dientamoeba fragilis*	[189]
*Mus musculus*	House mouse	*Enterocytozoon bieneusi*	[190]
		*C. hepaticum*	[191]
		*C. parvum*	[183]
		*H. nana*	[192]
		*H. diminuta, S. muris, T. taeniaeformis*	[193]
		*Leishmania major*	[194]
		*A.s cantonensis*	[195]
		*H. taichui*	[196]
		*T. gondii*	[197]
		*T. canis*	[198]
		*E. multilocularis*	[199]
*Rattus tanezumi* Temminck	Asian house rats	*A. cantonensis, Hymenolepis* spp., *T. taeniaeformis*	[200]
		*Syphacia muris, H. diminuta*	[201]
		*T. gondii*	[202]
		*C. hepaticum*	[203]
		*B. microti*	[98]
		*Artyfechinostomum malayanum, Echinostoma ilocanum, E. lindoense, H. nana, M. moniliformis, P. westermani, Plagiorchis muris, P. philippinensis, P. potamonides, Raillietina madagascariensis, S. japonicum, T. taeniaeformis*	[181]
*Rattus exulans*	Pacific rats	*S. japonicum*	[182]
		*H. diminuta, H. nana, M. moniliformis, Raillietina* spp.	[204]
		*A. cantonensis*	[19]
		*T. gondii*	[205]
*Ondatra zibethicus*	Musk rat	*E. multilocularis*	[206]
		*Taenia martis, T. taeniaeformis*	[207]
		*C. hepatica*	[208]
		*C. parvum*	[183]
*Myocastor coypus*	Nutria	*C. parvum*	[183]
		*T. gondii*	[209]
		*E. multilocularis*	[206]
		*F. hepatica*	[210]
		*Strongyloides myopotami*	[211]
		*C. hepatica*	[212]
*Sciurus vularis exalbidus*	Red squirrek	*T. gondii*	[213]
		*C. hepaticum*	[203]
		*C. parvum*	[166]
*Callosciurus erythraeus*	Red-bellied squirrel	*Hymenolepis* sp.	[214]
		*E. bieneusi*	[164]
		*A. cantonensis*	[215]
*Macaca fascicularis*		*C. parvum*	[216]
		*Plasmodium knowlesi*	[200, 217]
		*E. histolytica*	[218]
		*Endolimax nana, Oesophagostomum*	[219]
		*Sarcocystis* sp.	[220]
		*Strongyloides fulleborni*	[221]
		*H. nana, Strongyloides* spp.	[222]
		*Armillifer agkistrodontis*	[223]
		*G. duodenalis*	[224]
		*T. trichiura*	[225]
		*E. multilocularis*	[226]

#### Prevention and control

The invasion of alien species becomes much more common with predictable developing international communications and inevitable globalization [107]. Considering the great damage it may cause [108], the Chinese government has focused on the administration, international cooperation, fundamental researches and public awareness to cope with the problems [109]. The Ministry of Agriculture (MOA) has established the institution for administration of invasive alien species and a center for management of invasive alien species. MOA also organized to draw up the *Measures for Management of IAS*, rectified the *Contingency Plans for Emergency of Alien Species Invasion*, perfect the database of IAS in China and complete the prevention and control system. The Chinese government has put up some relevant legislation including *Law of the RPC on the Entry and Exit Animal and Plant Quarantine against the IAS* in 1996 [110], and the science funding regarding associated programs are booming as well [109].

However, the Chinese government should also issue more regulations to complete the legislation system toward the IAS and the current regulations should be enforced strictly. Surveillance and prevention system should be improved, and some cross-industry obligatory institutions should be established to integrate related work to fulfill the administration. The propagation of science knowledge should also be intensified to raise public awareness of the IAS.

For those species introduced deliberately as food material, the China Food and Drug Administration (CFDA) is supposed to take rigid strategy to restrict the introduction and make rigorous standard to ensure the safety and harmlessness of the species. For those IAS which entered China coincidentally, related institutions at the national border should intensify the surveillance and monitor system to prevent the invasion of unwanted species. For some IAS which have the potential to cause or has already caused enormous economic, environmental or public health problems, some eradication steps are supposed to be considered to prevent further loss.

## Conclusions

It can be predicted that the IAS issue may become a progressively important problem globally with the development of society and booming international communication. The government should raise awareness of the possible destructions the IAS may cause and take some measures in advance to prevent possible loss.

Apart from IAS’s direct damage to the ecology and economy, its capability as the vector to transmit parasitic disease should arouse public awareness. In China, as mentioned above, many species may play an important role in the transmission of some specific diseases, but the relative researches are rather scarce which suggests insufficient emphasis regarding such an issue. The risk of the parasites carried by the IAS is vastly underestimated. With various climates and multiple landforms, China is suitable for numerous IAS and parasites to settle down. However, few associated reports or researches can be found in China, as most case reports of zoonotic parasites carried by invasive alien species in China were listed in Table 7. More efforts about associated problems should be paid in both researching and administration. For those species which has already caused some zoonotic diseases, compulsory measures to control the species should be conducted to ensure the safety of public and some eradication methods ought to be considered for some more dangerous disease. For those species capable of transmitting parasites with no specific case reported yet, the screening surveys and control measures should be strictly fulfilled periodically to guarantee the safety of those species.

**Table 7:** Reported distribution and case reports of zoonotic parasites carried by invasive alien species in China

Parasites	Location of report	Reported local invasive alien host	Infection rate in investigated population	Number of Case reports	Reference
*Angiostrongylus cantonensis*	Yunnan, Guangxi, Hainan, Guangdong, Fujian, Zhejiang, Jiangsu, Beijing, Tianjin, Liaoning, Heilongjiang.	*Rattus rattus, R. norvegicus, Pomacea canaliculate, Achatina fulica*	–	334	[34, 96]
*Paragonimus westermani*	Hunan, Liaoning, Jilin, Heilongjiang, Sichuan, Yunnan, Shanxi, Zhejiang, Henan, Hubei, Hebei, Shaanxi, Gansu, Anhui, Guizhou, Guangdong, Hainan, Jiangxi, Fujian	*Procambarus clarkii*	1.71%	–	[47, 48]
*Haplorchis taichui*	Guangxi	*Cyprinus carpio, C. auratus*	24.3–90.5% (in people with a habit of consuming raw fish)	–	[70]
*Clonorchis sinensis*	Guangdong, Guangxi, Heilongjiang, Jilin, Liaoning, Hunan, Chongqing, Jiangsu, Anhui, Fujian, Hainan, Tianjin, Sichuan, Henan, Shandong, Hubei, Guizhou, Xinjiang, Hebei, Jiangxi, Shanghai, Shanxi, Beijing, Yunnan, Shanxi, Gansu, Zhejiang	*C. auratus, C carpio, O. mossambica*	2.4% (about 12.5 million people)	–	[73–75]
*Diphyllobothrium latum*	Taiwan, Heilongjiang, Jilin	*Oncorhynchus mykiss, Salvelinus forntinalis, Stizostedion vitreum, Perca flavescens*	–	16	[77]
*Gnathostoma* spp.	Zhejiang, Jiangsu, Anhui, Hunan, Hubei, Shandong, Henan, Jiangxi, Guangdong, Hainan, Taiwan, Fujian, Shanghai, Jilin, Heilongjiang, Xinjiang	*C. carpio, C. auratus*	–	More than 50	[79]
*Babesia microti*	Chongqing, Yunnan, Inner Mongolia, Taiwan, Zhejiang, Shandong, Shanxi, Xinjiang	*R. tanezumi, R. norvegicus*	–	27	[98]
*Echinococcus multilocularis*	northwest China, mainly Xinjiang, Ningxia, Gansu, Sichuan and Qinghai	*R. norvegicus, M. musculus*	–	Over 1000	[100–102]
*Capillaria hepatica*	–	*R. norvegicus, M. musculus*	–	3	[103, 104]

- Not applicable

## Additional file

Additional file 1: Multilingual abstracts in the five official working languages of the United Nations. (PDF 602 kb)
