# Supplementary material for: Zoonotic parasites carried by invasive alien species in China
Source: Infect Dis Poverty. 2019 Jan 9;8:2. doi: 10.1186/s40249-018-0512-6 (PMC6325848; doi:10.1186/s40249-018-0512-6)
Supplement: Supplementary file 1 — Multilingual abstracts in the five official working languages of the United Nations. (PDF 602 kb) [file 40249_2018_512_MOESM1_ESM.pdf]

Translation of the abstract into the five official working languages of the United Nations

الطفيليات الحيوانية المصدر التي تنقلها الأنواع الغريبة الغازية في الصين

شياو-تينغ لو ، كيو-يون غو ، يانين ليمبانون ، لان غوي سونج ، زونغ-داو وو ، كامولنيتر أوكانوراك ، زهي يو إل في

الملخص
خلفية الموضوع: قد تؤدي الأنواع الغريبة الغازية إلى أزمة بيئية واقتصادية كبيرة بسبب قدرتها القوية على احتلال المكانة البيولوجية للأنواع المحلية وتغيير النظام البيئي للمنطقة الغازية. ومع ذلك، فقد تم إهمال قدرتها على العمل كنواقل لبعض مسببات الأمراض الحيوانية المحددة، وخاصة الطفيليات. وبالتالي، فإن الضرر الذي قد يسببه قد تم التقليل منه بشكل كبير في هذا الجانب، وهو في الواقع مشكلة صحية عامة مهمة. تهدف هذه الورقة إلى مناقشة الوضع الحالي للطفيليات الحيوانية التي تنقلها الأنواع الغريبة الغازية في الصين.
الفكرة العامة يلخص هذا الاستعراض الطفيليات الحيوانية التي تم الأبلاغ عنها والتي تنقلها الأنواع الغريبة الغازية في الصين على أساس قاعدة بيانات الأنواع الغريبة الغازية في الصين. نحن نلخص انتشارها، وتهديدها لصحة الإنسان، والحالات المبلغ عنها ذات الصلة، وأدوار الأنواع الغريبة الغازية في دورة حياة هذه الطفيليات، وتاريخ الغزو لبعض الأنواع الغريبة الغازية. علاوة على ذلك، نلخص الحالة الحالية للوقاية والسيطرة على الأنواع الغريبة الغازية في الصين، ونناقش حول الحاجة الملحة وعديد من الاستراتيجيات الممكنة لمنع ومكافحة هذه الأمراض الحيوانية المنشأ في ظل ازدهار الاتصالات الدولية والعولمة التي لا مفر منها.
الاستنتاجات: إن المعلومات عن الطفيليات الحيوانية المصدر التي تنقلها الأنواع الغريبة الغازية لا في الصين أو في جميع أنحاء العالم، خاصة تقارير الحالات ذات الصلة محدودة بسبب إهمال طويل الأمد ونقص الرصد. ويستلزم التقليل من أضرارها إيلاء المزيد من الاهتمام للمراقبة والتحكم، وينبغي اتخاذ تدابير إلزامية للتحكم في الأنواع الغريبة الغازية التي تحمل الطفيليات الحيوانية.

Translated from English version into Arabic by Mohamed Shawkat, proofread by Heba Kandel, through

TRANSLATORS
WITHOUT BORDERS

中国入侵生物携带的人畜共患寄生虫

朱广李, 唐熠阳, Yanin Limpanont, 吴忠道, 李坚, 吕志跃

摘要
引言：入侵生物通过其竞争当地物种的生态位、改变入侵地生态系统的强大能力，对当地生态环境造成严重威胁。然而，其携带特定人畜共患病病原体的潜在风险（尤其是携带寄生虫的风险）长期未引起足够重视。入侵生物在这一方面导致的危害被明显低估，并对公共卫生安全造成了极大的威胁。本文拟对携带人兽共患寄生虫的中国入侵生物的现状进行概述。
主要内容：基于中国外来入侵物种数据库，本文对已有报道的中国入侵生物携带的人畜共患寄生虫进行综述，介绍了这些寄生虫的流行情况、对人类健康的危害、病例报告、入侵生物在其虫体生活史中的作用及部分入侵生物的入侵史。此外，本文还描述了中国防控入侵生物的现状，强调当今全球化和国际交流日益频繁的背景下防控这些人畜共患病的重要意义与紧迫性，并提出了可行的备选方案。

结论：由于长期忽视和监管缺失，中国乃至全球入侵生物携带人畜共患寄生虫的资料相当匮乏。医疗卫生部门应投入更多资源和采取更多措施进行入侵生物及其携带寄生虫的监管和防控。

Translated from English version into Chinese by Zhi-Yue Lv, Guang-Li Zhu, Yi-Yang Tang

Les zoonoses portées par des espèces exotiques envahissantes en Chine

Guang-Li Zhu, Yi-Yang Tang, Yanin Limpanont, Zhong-Dao Wu, Jian Li, Zhi-Yue Lv

Résumé

Contexte: Les espèces exotiques envahissantes peuvent provoquer de graves crises écologiques et économiques en raison de leur forte capacité à occuper les niches écologiques des espèces indigènes et à modifier l'écosystème de la zone envahie. On a cependant négligé leur capacité à servir de vecteur à certains agents pathogènes zoonotiques, en particulier à des parasites. Les dégâts qu'elles peuvent causer ont donc été largement sous-estimés de ce point de vue et un problème de sante publique important a ainsi été négligé. Le présent article dresse un tour d'horizon des zoonoses portées par les espèces exotiques envahissantes en Chine.

Discussion: La présente revue recense les zoonoses portées par des espèces exotiques signalées en Chine, d'après la base de données nationale des espèces exotiques envahissantes. Nous synthétisons leur prévalence, leur dangerosité pour la santé humaine, les cas signalés, le rôle des espèces exotiques envahissantes dans le cycle de vie de ces parasites et l'histoire de l'invasion par certaines espèces exotiques. En outre, nous résumons l'état actuel de la prévention et de la lutte contre les espèces exotiques envahissantes en Chine et abordons la question de l'urgence et des stratégies envisageables pour prévenir et contrôler ces zoonoses dans un contexte d'intensification des échanges internationaux et de mondialisation inéluctable.

Conclusions: Que ce soit en Chine ou ailleurs dans le monde, les informations concernant les zoonoses portées par des espèces exotiques envahissantes, et en particulier les rapports de cas correspondants, sont insuffisantes en raison d'une négligence et d'un manque de surveillance de longue date. Parce que les dommages qu'elles peuvent causer ont été sous-estimés, il faut à présent intensifier la surveillance et la lutte, et des mesures contraignantes doivent être prises afin de luter contre les espèces exotiques envahissantes porteuses de zoonoses.

Translated from English version into French by G. Lecuyer, proofread by Suzanne Assenat, through

TRANSLATORS
WITHOUT BORDERS

Зоонозные паразиты, переносимые инвазивными чужеродными видами в Китае

Гуан-Ли Чжоу, И-Ян Тан, Янин Лимпанон, Чжун-Дао Ву, Цзянь Ли, Чжи-Уэ Люй

Аннотация

Предпосылки исследования: Инвазивные чужеродные виды могут вызывать серьёзный экологический и экономический кризис благодаря своей мощной способности занимать биологическую нишу местных видов и изменять экосистему захваченного региона. Однако их способности служить векторами определенных зоонозных патогенов, особенно паразитов, не уделяется должного внимания. Поэтому потенциальный вред, который они могут нанести, серьёзно недооценивается в данном аспекте, и это является важной проблемой здравоохранения. Цель настоящей работы — обсудить текущий статус зоонозных паразитов, переносимых инвазивными чужеродными видами в Китае.

Основная часть: В данном обзоре кратко изложена информация о зоонозных паразитах, переносимых инвазивными чужеродными видами в Китае, на основе китайской Базы данных инвазивных чужеродных видов. Мы подытоживаем данные относительно их распространённости, угрозы для здоровья человека на основе зарегистрированных случаев, роли инвазивных чужеродных видов в жизненном цикле данных паразитов, а также истории вторжения некоторых инвазивных чужеродных видов. Помимо этого, мы суммируем текущее состояние профилактики и контроля инвазивных чужеродных видов в Китае, а также обсуждаем неотложность и ряд практически осуществимых стратегий профилактики и контроля данных зооноз в контексте международных коммуникаций и неизбежной глобализации.

Выводы: Информация о зоонозных паразитах, переносимых инвазивными чужеродными видами в Китае и во всём мире, особенно о зарегистрированных случаях, ограничена из-за длительного игнорирования и отсутствия мониторинга. Недооценка наносимого ими ущерба требует большего внимания к мониторингу, контролю и обязательным мерам, которые нужно принять для контроля инвазивных чужеродных видов, переносящих зоонозных паразитов.

Translated from English version into Russian by Liudmila Tomanek, proofread by Olga Madiar, through

TRANSLATORS WITHOUT BORDERS

Parásitos zoonóticos portados por especies exóticas invasoras en China

Xiao-Ting Lu, Qiu-Yun Gu, Yanin Limpanont, Lan-Gui Song, Zhong-Dao Wu, Kamolnetr Okanurak, Zhi-Yue Lv

Resumen

Antecedentes: Las especies exóticas invasoras pueden causar graves crisis medioambientales y económicas debido a su gran capacidad para ocupar el nicho ecológico de las especies nativas y alterar el ecosistema de la zona invadida. Sin embargo, apenas se ha prestado atención a su capacidad para actuar como vectores de determinados patógenos zoonóticos, especialmente parásitos. Por consiguiente, se han subestimado con creces los daños que pueden producir en este sentido, lo cual

constituye un grave problema de salud pública. Este artículo tiene como objetivo analizar la situación actual respecto a los parásitos zoonóticos portados por especies exóticas invasoras en China.

Cuerpo del texto: En este análisis presentaremos una síntesis de los parásitos zoonóticos transmitidos por especies exóticas invasoras registrados en China tomando como referencia la Base de datos de especies exóticas invasoras de China. Ofreceremos un resumen sobre su prevalencia, la amenaza que suponen para la salud humana, los casos registrados relacionados, el papel de la especie exótica invasora en el ciclo de vida de estos parásitos y el historial invasor de algunas especies exóticas. Además, resumiremos la situación actual en materia de prevención y control de especies exóticas invasoras en China y analizaremos varias estrategias factibles que se deben adoptar urgentemente para prevenir y controlar estas zoonosis en la época del auge de las comunicaciones internacionales florecientes y la inevitable globalización.

Conclusiones: La información disponible sobre los parásitos zoonóticos transmitidos por especies exóticas invasoras, especialmente sobre los casos clínicos relacionados, es limitada, tanto en China como a nivel mundial, debido a una carencia histórica de estudios al respecto y a la falta de seguimiento. Dado que se han subestimado los daños que estos pueden causar, es preciso que se realicen más seguimientos y controles. Además, se deberían tomar medidas obligatorias para controlar a las especies exóticas invasoras portadoras de parásitos zoonóticos.

Translated from English version into Spanish by Noelia Bernardez, proofread by Ribcamar, through

TRANSLATORS
WITHOUT BORDERS
